# Scientific dissemination in CSP: importance, advances, and challenges

**DOI:** 10.1590/0102-311XEN206824

**Published:** 2025-01-13

**Authors:** Luciana Dias de Lima, Clara Guimarães, Carolina Ribeiro, Luciana Correia Alves, Marilia Sá Carvalho

**Affiliations:** 1 Escola Nacional de Saúde Pública Sergio Arouca, Fundação Oswaldo Cruz, Rio de Janeiro, Brasil.; 2 Instituto de Filosofia e Ciências Humanas, Universidade Estadual de Campinas, Campinas, Brasil.; 3 Programa de Computação Científica, Fundação Oswaldo Cruz, Rio de Janeiro, Brasil.

“Doing science” system refers to the set of processes, institutions, practices, and actors involved in the production, validation, and dissemination of scientific knowledge. It includes the entire process, from hypothesizing research questions to implementing its results in society. Scientific dissemination is increasingly recognized as an essential component of this system.

Such appreciation is partially due to the so-called “infodemic”, boosted during the COVID-19 pandemic, even in environments dedicated to scientific communication. In this context, the excess of information becomes noise, generating discredit for science and scientists, with deleterious effects on population health.

The drop in vaccination coverage, for example, was identified in 2019 as one of the 10 issues to be faced in the context of global health ^1^. Such decline was generated and intensified by anti-vaccine movements, which fuel concerns about vaccination safety and efficacy, and create a false contradiction between individual freedom and the collective good ^2^. The pandemic has highlighted the urgency of strengthening science divulgation , aiming at making research results accessible, especially in face of anti-science movements ^3^.

Journals play a fundamental role in communicating the science being produced. We do not refer to predatory journals, which publish under payment, regardless of the data reliability. Peer review is still the best evaluation method, even considering its limitations ^4^ ^,^ ^5^. This capital forms the foundation for scientific dissemination of journals, which is the cornerstone of CSP’s proposal.

Thus, CSP aligns itself with the Oswaldo Cruz Foundation’s Scientific Dissemination Policy ^6^, that emphasizes the importance of sharing scientific knowledge and of its incorporation by society, which by definition should have the right to access such knowledge. This policy is in line with open science practices: both share the goal of democratizing knowledge and expanding access to information. While open access to knowledge is concerned with ensuring infrastructure and spreading the values of free access ^7^, scientific communication is responsible for communicating and translating such knowledge, contributing to a more informed and engaged society. Together, they reinforce the role of science as a noncommercial public good, a principle that guides CSP’s editorial policy.

In 2018, CSP committed itself to expand the dissemination of its publications, investing in different fronts of scientific communication: social networks, contact with the press, and audiovisual production ^8^. By the end of 2024, the journal can be found on Facebook, Instagram, and X (Twitter). However, the algorithms of these platforms tend to create content bubbles, based on likes and shares, making it difficult to expand the outreach beyond “peers” ^9^. Additionally, CSP decided to move away from X, due to its owner’s attacks against the Brazilian Democratic Rule of Law ^10^.

Due to the COVID-19 pandemic, dissemination efforts were intensified and the *Interview with Authors* show was created, aiming to quickly disseminate science published under the fast-track system. The program, available on YouTube and on various audio platforms, features monthly interviews with authors of articles selected by the Board of Editors. By November, the podcast had more than 4,000 plays and surpassed 20,000 views on YouTube.

CSP also has three major journalistic partners: Agência Bori, a service that supports press coverage nationwide in the light of scientific evidence ^11^; Outra Saúde, specialized in articles and reports in health ^12^; and Nexo Políticas Públicas - academic-journalistic platform of Nexo Journal ^13^. In total, 75 articles were published through these collaborations.

We understand that the readers of these outlets are often familiar with health and science. However, these partnerships significantly boost the dissemination of papers, since over 400 articles have been republished in recent years by national outlets, such as Agência Brasil, Folha de S.Paulo, and O Globo.

Scientific dissemination in CSP seeks to attract new readers and expand the audience interested in science and health. However, assessing the real impact of the different media is challenging, especially in face of constant changes and opacity in social media algorithms.

It is necessary to question to what extent the journal really expands its audience on these platforms or if, on the contrary, it ends up favoring the interests of the networks themselves in directing content to already established profiles ^14^. Thus, the debate about social networks effectiveness in expanding science outreach persists. Overall, despite the still limited knowledge on the concrete impact of this form of scientific dissemination, the presence in these media is strategic, as they represent spaces widely frequented by younger audience, whether future researchers or not. Therefore, it is believed that this interaction contributes to scientific literacy, integrating scientific knowledge into the daily practices of this generation in formation, which is fundamental for the development of an informed and critical society ^15^.

After five years of continuous investments in scientific dissemination, CSP now seeks to evaluate its outreach and impact, investigating questions such as: who are the readers our journal reaches on social networks? What is the volume of accesses to the articles originating from each platform? Who is the audience effectively reached? In the year the journal celebrates its 40th anniversary, these issues are being carefully analyzed, seeking to improve strategies and consolidate the CSP’s role in scientific dissemination.

We end this special year of celebration with gratitude to all who have collaborated with the journal in its trajectory, and we renew our commitment to its mission: to disseminate reliable scientific knowledge, overcome academic isolation, strengthen dialogue, and promote the advancement of science for the benefit of society. Happy holidays!
