# Supplementary material for: Scientific dissemination in CSP: importance, advances, and challenges
Source: Cad Saude Publica. 2025 Jan 13;40(12):e00206824. doi: 10.1590/0102-311XEN206824 (PMC11729400; doi:10.1590/0102-311XEN206824)
Supplement: Translation

# Translation: Divulgação científica em CSP: importância, avanços e desafios

**DOI:** 10.1590/0102-311XPT206824

**Authors:** Luciana Dias de Lima, Clara Guimarães, Carolina Ribeiro, Luciana Correia Alves, Marilia Sá Carvalho

**Affiliations:** 1 Escola Nacional de Saúde Pública Sergio Arouca, Fundação Oswaldo Cruz, Rio de Janeiro, Brasil.; 2 Instituto de Filosofia e Ciências Humanas, Universidade Estadual de Campinas, Campinas, Brasil.; 3 Programa de Computação Científica, Fundação Oswaldo Cruz, Rio de Janeiro, Brasil.

O sistema “fazer ciência” refere-se ao conjunto de processos, instituições, práticas e atores envolvidos na produção, na validação e na disseminação do conhecimento científico. Ele abrange desde a formulação de perguntas de pesquisa até a aplicação dos resultados na sociedade. A divulgação científica é cada vez mais reconhecida como componente essencial desse sistema.

Em parte, essa valorização se deve à chamada “infodemia”, acentuada durante a pandemia de COVID-19, mesmo em ambientes dedicados à comunicação científica. Nesse contexto, o excesso de informação se torna ruído, gerando descrédito para a ciência e os cientistas, com efeitos deletérios para a saúde da população.

A queda nas coberturas vacinais, por exemplo, já foi identificada como uma das dez questões a serem enfrentadas no âmbito da saúde global em 2019 ^1^. Essa queda foi gerada e intensificada por movimentos ativamente antivacina, que incentivam os receios sobre segurança e eficácia, e criam uma falsa contradição entre liberdade individual e bem coletivo ^2^. A pandemia destacou a urgência de fortalecer a comunicação pública da ciência, visando tornar acessiveis os resultados de pesquisas, especialmente diante de movimentos anticiência ^3^.

Os periódicos têm papel fundamental na comunicação da ciência produzida. Aqui não estamos falando de periódicos predatórios, que na verdade publicam qualquer coisa, basta pagar. A revisão por pares ainda é o melhor mecanismo de avaliação, mesmo considerando suas limitações ^4^ ^,^ ^5^. A partir desse capital é que se estabelece o potencial de divulgação científica dos periódicos, base da proposta de CSP.

Com isso, CSP se alinha à Política de Divulgação Científica da Fundação Oswaldo Cruz ^6^, que ressalta a importância do compartilhamento do conhecimento científico e de sua incorporação pela sociedade, que por definição deve ter o direito de acesso a tal conhecimento. Essa política vai ao encontro das práticas de ciência aberta. Ambas compartilham o objetivo de democratizar o conhecimento e ampliar o acesso à informação. Enquanto o acesso aberto ao conhecimento se preocupa em garantir infraestrutura e difundir os valores do acesso livre ^7^, a divulgação científica se encarrega de comunicar e traduzir esse conhecimento, contribuindo para uma sociedade mais informada e engajada. Juntas, elas reforçam o papel da ciência como bem público não comercial, princípio que orienta a política editorial de CSP.

Em 2018, CSP assumiu o compromisso de ampliar a divulgação de seus artigos, investindo em diferentes frentes de comunicação científica: redes sociais, contato com a imprensa e produção audiovisual ^8^. Ao final de 2024, a revista está presente no Facebook, Instagram e X (antigo Twitter). No entanto, os algoritmos dessas plataformas tendem a criar bolhas de conteúdo, baseadas em curtidas e compartilhamentos, dificultando a ampliação do alcance para além dos “pares” ^9^. Além disso, CSP decidiu se afastar do X, devido aos ataques de seu proprietário contra o Estado Democrático de Direito brasileiro ^10^.

Com a pandemia de COVID-19, os esforços de divulgação foram intensificados e o programa *Entrevista com Autores* foi criado, com o objetivo de disseminar rapidamente a ciência publicada em regime de *fast-track*. O programa, disponível no YouTube e em diversas plataformas de áudio, apresenta entrevistas mensais com autores de artigos selecionados pela Editoria. Até o mês de novembro, o *podcast* contava com mais de 4 mil *plays* e ultrapassava 20 mil visualizações no YouTube.

CSP conta ainda com três grandes parceiros jornalísticos: a Agência Bori, serviço que apoia a cobertura da imprensa de todo o país à luz de evidências científicas ^11^; Outra Saúde, especializada em matérias e reportagens na área da saúde ^12^; e Nexo Políticas Públicas, plataforma acadêmico-jornalística do Nexo Jornal ^13^. Ao todo, 75 matérias foram publicadas por meio dessas colaborações.

Compreendemos que os leitores desses veículos são, muitas vezes, pessoas já familiarizadas com a saúde e a ciência. Entretanto, essas parcerias impulsionam significativamente a divulgação dos manuscritos, uma vez que mais de 400 matérias foram republicadas ao longo desses anos por veículos de alcance nacional, como Agência Brasil, Folha de S.Paulo e O Globo.

A divulgação científica em CSP busca atrair novos leitores e ampliar o público interessado em ciência e saúde. No entanto, avaliar o impacto real das diferentes mídias utilizadas é um desafio, especialmente diante das constantes mudanças e opacidade nos algoritmos das redes sociais.

É necessário questionar até que ponto a revista realmente amplia seu público nessas plataformas ou se, ao contrário, acaba favorecendo os interesses das próprias redes em direcionar conteúdo a perfis já estabelecidos ^14^. Persiste, assim, o debate sobre a efetividade das redes sociais em ampliar o alcance da ciência. De modo geral, apesar do ainda limitado conhecimento sobre o impacto concreto dessa forma de divulgação científica, a presença nesses meios é estratégica, pois representam espaços amplamente frequentados por jovens, sejam futuros pesquisadores ou não. Dessa forma, acredita-se que essa interação contribui para o letramento científico, integrando o conhecimento científico às práticas cotidianas dessa geração em formação, o que se revela fundamental para o desenvolvimento de uma sociedade informada e crítica ^15^.

Após cinco anos de investimentos contínuos em divulgação científica, CSP busca agora avaliar seu alcance e impacto, investigando questões como: quem são os leitores alcançados pelo periódico nas redes sociais? Qual é o volume de acessos aos artigos originados a partir de cada plataforma? Quem é, de fato, o público efetivamente atingido? No ano em que a revista comemora 40 anos, essas questões estão sendo analisadas com atenção, buscando aperfeiçoar estratégias e consolidar o papel de CSP na divulgação científica.

Encerramos este ano especial de celebração com gratidão a todos que colaboraram com o periódico em sua trajetória, e renovamos nosso compromisso com sua missão: disseminar conhecimento científico de qualidade, superar o isolamento acadêmico, fortalecer o diálogo e promover o avanço da ciência em prol da sociedade. Boas festas!

# Translation: Divulgación científica en CSP: importancia, avances y desafíos

**DOI:** 10.1590/0102-311XES206824

**Authors:** Luciana Dias de Lima, Clara Guimarães, Carolina Ribeiro, Luciana Correia Alves, Marilia Sá Carvalho

**Affiliations:** 1 Escola Nacional de Saúde Pública Sergio Arouca, Fundação Oswaldo Cruz, Rio de Janeiro, Brasil.; 2 Instituto de Filosofia e Ciências Humanas, Universidade Estadual de Campinas, Campinas, Brasil.; 3 Programa de Computação Científica, Fundação Oswaldo Cruz, Rio de Janeiro, Brasil.

El sistema “hacer ciencia” se refiere al conjunto de procesos, instituciones, prácticas y actores que participan en la producción, validación y difusión del conocimiento científico. Abarca desde la formulación de preguntas de investigación hasta la aplicación de los resultados en la sociedad. La divulgación científica es, cada vez más, reconocida como un componente esencial de este sistema.

En parte, esta valorización se debe a la llamada “infodemia”, acentuada durante la pandemia de COVID-19, incluso en entornos dedicados a la comunicación científica. En este contexto, el exceso de información se convierte en ruido y genera descrédito para la ciencia y los científicos, con efectos nocivos para la salud de la población.

La reducción de la cobertura de vacunación, por ejemplo, ya ha sido identificada como una de las diez cuestiones que afrontar en el contexto de la salud mundial en el 2019 ^1^. Esta reducción fue generada e intensificada por movimientos activos contra las vacunas, que fomentan temores sobre la seguridad y la eficacia, y, crean una falsa contradicción entre la libertad individual y el bien colectivo ^2^. La pandemia puso de relieve la urgencia de fortalecer la comunicación pública de la ciencia, con el objetivo de hacer accesibles los resultados de investigaciones, especialmente frente a los movimientos anticiencia ^3^.

Las revistas desempeñan un papel fundamental en la comunicación de la ciencia producida. Aquí no estamos hablando de revistas depredadoras, que en realidad publican cualquier cosa, con solo pagar. La revisión por pares sigue siendo el mejor mecanismo de evaluación, incluso teniendo en cuenta sus limitaciones ^4^ ^,^ ^5^. Es con base en este capital que se establece el potencial de divulgación científica de las revistas, base de la propuesta de CSP.

Con ello, CSP se alinea con la Política de Divulgación Científica de la Fundación Oswaldo Cruz ^6^ lo que resalta la importancia de compartir el conocimiento científico y de su incorporación por parte de la sociedad, que por definición debe tener derecho a acceder a dicho conocimiento. Esta política está en consonancia con las prácticas de ciencia abierta. Ambos comparten el objetivo de democratizar el conocimiento y ampliar el acceso a la información. Mientras que el acceso abierto al conocimiento se preocupa por garantizar la infraestructura y difundir los valores del libre acceso ^7^, la divulgación científica se encarga de comunicar y traducir este conocimiento, contribuyendo a una sociedad más informada y comprometida. Juntas, refuerzan el papel de la ciencia como bien público no comercial, un principio que guía la política editorial de CSP.

En el 2018, CSP se comprometió a ampliar la difusión de sus artículos, invirtiendo en diferentes frentes de comunicación científica: redes sociales, contacto con la prensa y producción audiovisual ^8^. A finales del 2024, la revista está presente en Facebook, Instagram y X (Twitter). Sin embargo, los algoritmos de estas plataformas tienden a crear burbujas de contenido, basadas en la cantidad de interacciones “me gusta” y “compartir”, lo que dificulta ampliar el alcance más allá de los “pares” ^9^. Además, CSP decidió alejarse de X, debido a los ataques de su propietario al Estado Democrático de Derecho brasileño ^10^.

Con la pandemia de COVID-19, se intensificaron los esfuerzos de difusión y se creó el programa *Entrevista con Autores*, con el objetivo de difundir rápidamente la ciencia publicada de forma *fast-track*. El programa, disponible en YouTube y en varias plataformas de audio, presenta entrevistas mensuales con autores de artículos seleccionados por el Consejo Editorial. Hasta noviembre, el *podcast* contaba con más de 4.000 reproducciones y superó las 20.000 reproducciones en YouTube.

CSP también cuenta con tres grandes socios periodísticos: Agência Bori, un servicio que apoya la cobertura de prensa en todo el país a la luz de la evidencia científica ^11^; Outra Saúde, especializada en artículos y reportajes en el área de la salud ^12^; y Nexo Políticas Públicas, una plataforma académico-periodística de Nexo Jornal ^13^. En total, se publicaron 75 artículos por medio de estas colaboraciones.

Entendemos que los lectores de estos vehículos suelen ser personas que ya están familiarizadas con la salud y la ciencia. Sin embargo, estas asociaciones impulsan significativamente la difusión de los manuscritos, una vez que a lo largo de los años se volvieron a publicar más de 400 artículos en vehículos de alcance nacional, como Agência Brasil, Folha de S.Paulo y O Globo.

La divulgación científica en CSP busca atraer nuevos lectores y ampliar el público interesado en ciencia y salud. Sin embargo, evaluar el impacto real de los diferentes medios utilizados es un desafío, especialmente ante los constantes cambios y la opacidad en los algoritmos de las redes sociales.

Cabe preguntarse hasta qué punto la revista realmente amplía su público en estas plataformas o si, por el contrario, termina por favorecer los intereses de las propias redes en dirigir contenidos a perfiles ya establecidos ^14^. Por lo tanto, persiste el debate sobre la efectividad de las redes sociales para ampliar el alcance de la ciencia. En general, a pesar del conocimiento aún limitado sobre el impacto concreto de esta forma de divulgación científica, la presencia en estos medios es estratégica, ya que representan espacios muy frecuentados por jóvenes, futuros investigadores o no. De esta manera, se cree que esta interacción contribuye a la alfabetización científica, integrando el conocimiento científico en las prácticas cotidianas de esta generación en formación, lo que resulta fundamental para el desarrollo de una sociedad informada y crítica ^15^.

Después de cinco años de inversión continua en divulgación científica, CSP ahora busca evaluar su alcance e impacto, investigando cuestiones como: ¿Quiénes son los lectores a los que llega la revista en las redes sociales? ¿Cuál es el volumen de acceso a los artículos provenientes de cada plataforma? ¿Quién es, de hecho, el público objetivo? En el año en que la revista celebra su 40 aniversario, estos temas están siendo analizados detenidamente, buscando mejorar estrategias y consolidar el papel de la CSP en la divulgación científica.

Cerramos este año especial de celebración expresando nuestra gratitud a todos los que colaboraron con la revista a lo largo de su trayectoria, y renovamos nuestro compromiso con su misión: difundir conocimiento científico de calidad, superar el aislamiento académico, fortalecer el diálogo y promover el avance de la ciencia a favor de la sociedad. ¡Felices fiestas!
